# Supplementary material for: Protection from α-Synuclein induced dopaminergic neurodegeneration by overexpression of the mitochondrial import receptor TOM20
Source: NPJ Parkinsons Dis. 2020 Dec 8;6:38. doi: 10.1038/s41531-020-00139-6 (PMC7722884; doi:10.1038/s41531-020-00139-6)
Supplement: Supplementary file 1 — Supplementary Figures [file 41531_2020_139_MOESM1_ESM.pdf]

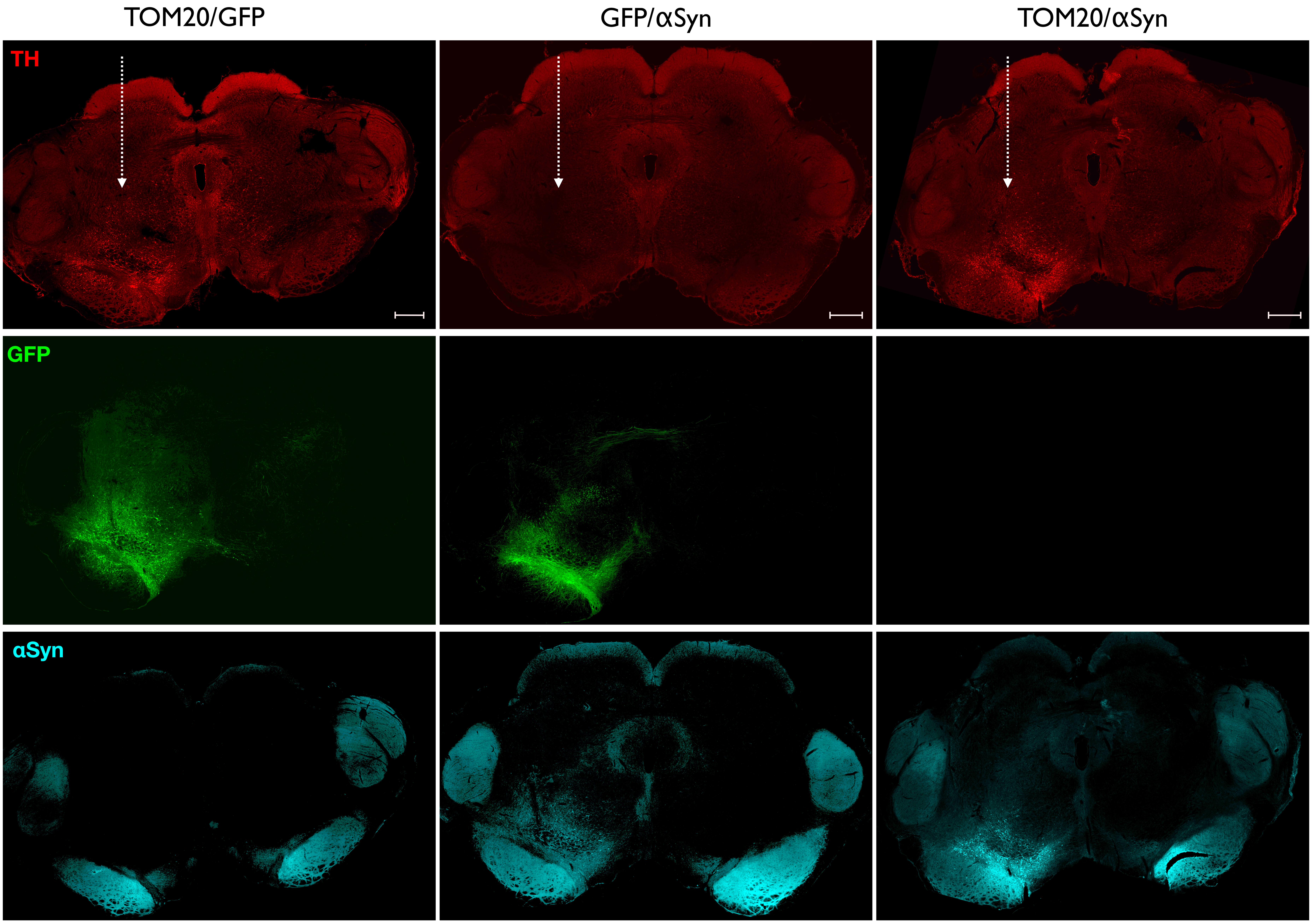

Supplemental Figure 1. Protein expression corresponding to viral vector injection in the rat ventral midbrain.

Coronal sections (35 μM) of rat brain tissue corresponding to the approximate Bregma -5.80 mm region targeted for viral injection. Total protein stains for TOM20 (red), GFP (green), and α-Synuclen (cyan) show both endogenous and vector-derived protein expression. Montage images reconstructed from 20x objective, scale bars 1mm, arrow indicates needle tract.

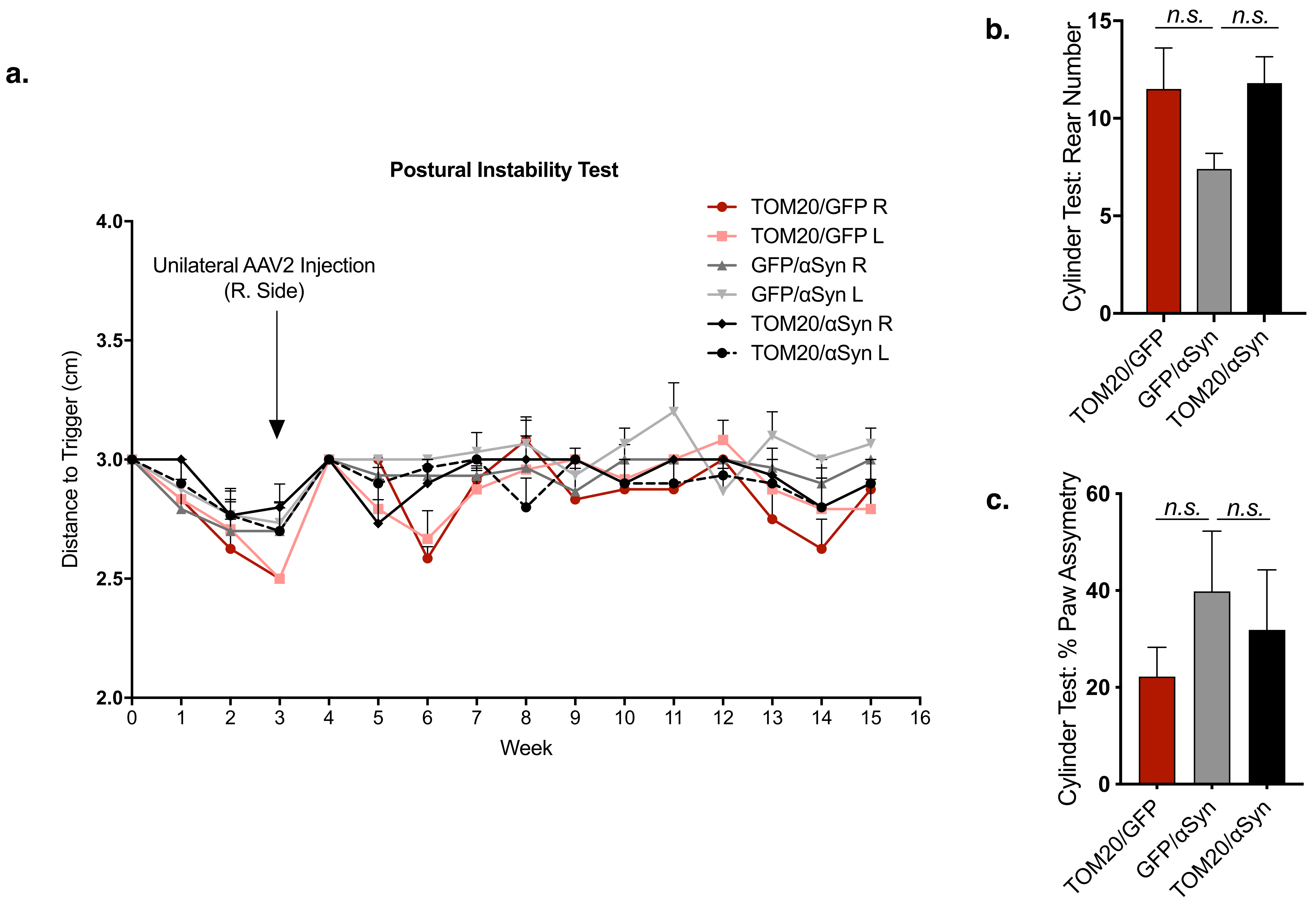

Supplemental Figure 2. AAV2-αSyn expression within the substantia nigra did not result in significant behavioral abnormalities.

A. The postural instability test (PIT) was performed at baseline (prior to vector infusion) and over the 12-week study timecourse. **B.** The average number of rearing movements from the cylinder test, performed at week 12. **C.** Percentage of paw asymmetry percent asymmetry from the cylinder test, calculated as [(% ipsilateral paw use – % contralateral paw use) / (% ipsilateral paw use + % contralateral paw use)] × 100.
