# Supplementary material for: Protection from α-Synuclein induced dopaminergic neurodegeneration by overexpression of the mitochondrial import receptor TOM20
Source: NPJ Parkinsons Dis. 2020 Dec 8;6:38. doi: 10.1038/s41531-020-00139-6 (PMC7722884; doi:10.1038/s41531-020-00139-6)
Supplement: Supplementary file 2 — Reporting Summary [file 41531_2020_139_MOESM2_ESM.pdf]

nature research

Corresponding author(s): J. Timothy Greenamyre

Last updated by author(s): 10/05/2020

Reporting Summary

Nature Research wishes to improve the reproducibility of the work that we publish. This form provides structure for consistency and transparency in reporting. For further information on Nature Research policies, see Authors & Referees and the Editorial Policy Checklist.

Statistics

For all statistical analyses, confirm that the following items are present in the figure legend, table legend, main text, or Methods section.

n/a	Confirmed	
☐	☒	The exact sample size (*n*) for each experimental group/condition, given as a discrete number and unit of measurement
☐	☒	A statement on whether measurements were taken from distinct samples or whether the same sample was measured repeatedly
☐	☒	The statistical test(s) used AND whether they are one- or two-sided<br>*Only common tests should be described solely by name; describe more complex techniques in the Methods section.*
☐	☒	A description of all covariates tested
☐	☒	A description of any assumptions or corrections, such as tests of normality and adjustment for multiple comparisons
☐	☒	A full description of the statistical parameters including central tendency (e.g. means) or other basic estimates (e.g. regression coefficient) AND variation (e.g. standard deviation) or associated estimates of uncertainty (e.g. confidence intervals)
☐	☒	For null hypothesis testing, the test statistic (e.g. *F*, *t*, *r*) with confidence intervals, effect sizes, degrees of freedom and *P* value noted<br>*Give P values as exact values whenever suitable.*
☒	☐	For Bayesian analysis, information on the choice of priors and Markov chain Monte Carlo settings
☒	☐	For hierarchical and complex designs, identification of the appropriate level for tests and full reporting of outcomes
☒	☐	Estimates of effect sizes (e.g. Cohen's *d*, Pearson's *r*), indicating how they were calculated

Our web collection on statistics for biologists contains articles on many of the points above.

Software and code

Policy information about availability of computer code

Data collection	Fluorescent immunohistochemical images were collected using an Olympus BX61 confocal microscope and Fluoview 1000 software (Melville, NY)
Data analysis	Confocal images were analyzed using Nikon NIS-Elements Advanced Research software (Version 4.5, Nikon, Melville, NY). Statistical analyses were carried out using GraphPad Prism software (V. 8.3.0).

For manuscripts utilizing custom algorithms or software that are central to the research but not yet described in published literature, software must be made available to editors/reviewers. We strongly encourage code deposition in a community repository (e.g. GitHub). See the Nature Research guidelines for submitting code & software for further information.

Data

Policy information about availability of data

All manuscripts must include a data availability statement. This statement should provide the following information, where applicable:

- Accession codes, unique identifiers, or web links for publicly available datasets
- A list of figures that have associated raw data
- A description of any restrictions on data availability

The data that support the findings of this study are available from the corresponding author on reasonable request.

Field-specific reporting

Please select the one below that is the best fit for your research. If you are not sure, read the appropriate sections before making your selection.

☒ Life sciences ☐ Behavioural & social sciences ☐ Ecological, evolutionary & environmental sciences

October 2018

Life sciences study design

All studies must disclose on these points even when the disclosure is negative.

Sample size	An a priori power analysis was conducted to determine the minimal number of animals required to achieve 20-40% variance of the mean, with a 95% power at α=0.05 using G*Power statistical software (N=5 per group)
Data exclusions	No data were excluded from analyses in this study.
Replication	Quantitative fluorescence measurements, the predominant endpoint measure for this study, were thoroughly monitored using standard operating imaging parameters to ensure that images contained no saturated pixels. For quantitative comparisons, all imaging parameters (e.g., laser power, exposure, pinhole) were held constant across specimens. Confocal images were analyzed using Nikon NIS-Elements Advanced Research software (Version 4.5, Nikon, Melville, NY), which is a semi-automated method designed to reduce researcher bias. A minimum of 6 images per tissue slice were analyzed per animal, averaging 9-15 neurons per 60-100x image (approximately 180 cells per animal, per histological stain). Replication attempts between tissue section from rats in the same treatment group were successful.
Randomization	Adult, male Lewis rats were received at the University of Pittsburgh DLAR facility and placed into single housing cages in preparation for surgery. Animals were chosen at random for treatment groups. Weights from each animal were recorded to ensure similar weight distribution between all groups. Rat weight distribution was within 50g. Following stereotactic surgery for viral vector delivery, and animal recovery, rats were coded using a 4-digit randomized code.
Blinding	Rats were coded using a 4-digit randomized code blinded to researchers carrying out analysis for the entirety of the study. Only one researcher had access to the codes, which were revealed after final statistical analyses were complete.

Reporting for specific materials, systems and methods

We require information from authors about some types of materials, experimental systems and methods used in many studies. Here, indicate whether each material, system or method listed is relevant to your study. If you are not sure if a list item applies to your research, read the appropriate section before selecting a response.

Materials & experimental systems

n/a	Involved in the study	
☐	☒	Antibodies
☒	☐	Eukaryotic cell lines
☒	☐	Palaeontology
☐	☒	Animals and other organisms
☒	☐	Human research participants
☒	☐	Clinical data

Methods

n/a	Involved in the study	
☒	☐	ChIP-seq
☒	☐	Flow cytometry
☒	☐	MRI-based neuroimaging

Antibodies

Antibodies used

Tyrosine hydroxylase AB1542 1:2000 EMD Millipore (Burlington, MA)
Human-α-Synuclein (LB509) Ab27766 1:1000 Abcam (Cambridge, MA)
COX-IV Ab16056 1:500 Abcam (Cambridge, MA)
GRP75/mtHSP70 Ab2799 1:500 Abcam (Cambridge, MA)
TOM20 (Ms) 612278 1:500 BD Biosciences (San Jose, CA)
α-Synuclein (total) 610787 1:500 BD Biosciences (San Jose, CA)
NDUFS3/OxPhos 459130 1:500 Thermo Fisher (Waltham, MA)
TOM20 (Rb) SC-11415 1:500 Santa Cruz Biotechnology (Santa Cruz, CA)
SDHA NBP1-71688 1:500 Novus Biologicals (Centennial, CO)

Validation

Tyrosine hydroxylase AB1542 Millipore QC Report: Routinely evaluated by Western Blot on mouse brain lysates. Western Blot Analysis: 1:1000 dilution of this lot detected tyrosine hydroxylase on 10 µg of mouse brain lysates.
Human-α-Synuclein (LB509) Ab 27766 Abcam QC Report: WB: Recombinant human Alpha-synuclein protein. Flow Cyt: PC12 (NGF differentiated) cells.
COX-IV Ab16056 Abcam QC Report: Detects a band of approximately 15 kDa (predicted molecular weight: 17 kDa). Rated for mitochondrial loading control.
GRP75/mtHSP70 Ab2799 Abcam QC Report: Staining of mtHSP 70 in DAP.3 cells results in a worm-like staining pattern, consistent with mitochondrial localization. Synthetic peptide corresponding to Mouse Grp75/MOT aa 661-679. Sequence: GSGSSGTGEQKEDQKEEKQ
TOM20 (Ms) 612278 BD Biosciences QC Report: Western blot (Routinely Tested), Immunofluorescence (Tested During Development).
α-Synuclein (total) 610787 BD Biosciences QC Report:Western blot (Routinely Tested), Immunofluorescence (Tested During Development) , Immunohistochemistry (Reported).
NDUFS3/OxPhos 459130 ThermoFisher QC Report: Isolated mitochondria from Human, Bovine Rat and Mouse hearts; Cultured

Human fibroblasts; HeLa cells; Human colon and cerebellum tissues as positive control.
TOM20 (Rb) SC-11415 Santa Cruz Biotechnology QC Report: Positive control, Western blot (routinely tested) in Jurkat cells produces a band at 20 kDa.
SDHA NBP1-71688 Novus QC Report: ICC/IF suitable. Western blot validated with a band observed at ~72 kDa. Control is NIH 3T3 Whole Cell Lysate.

Animals and other organisms

Policy information about studies involving animals; ARRIVE guidelines recommended for reporting animal research

Laboratory animals	Male, Lewis rats, 10 months old (Envigo, Indianapolis, IN)
Wild animals	Wild animals were not used in this study
Field-collected samples	Field samples were not used in this study
Ethics oversight	All experiments involving animal treatment and euthanasia were approved by the University of Pittsburgh Institutional Animal Care and Use Committee

Note that full information on the approval of the study protocol must also be provided in the manuscript.
